# Profil épidémio-clinique et séminal de l’homme consultant pour désir de procréation: état des lieux à Lubumbashi, en République Démocratique du Congo

**DOI:** 10.11604/pamj.2023.45.177.36977

**Published:** 2023-08-22

**Authors:** Jean Jimmy Kalfando Mwamba, Olivier Mukuku, Kumelundu Kasongo, Herman Kitoko Tamubango, Cynthia Mwenya Kibwe, Ignace Nday Tshikala, Jeannot Bakajika Kabue, Jean-Paul Nkenga Ilunga, Philémon Matumo Mumbere, Rivain Fefe Iteke, Joseph Bulanda Nsambi, Prosper Luhete Kakudji, Xavier Kalume Kinenkinda, Jean-Baptiste Kakoma

**Affiliations:** 1 Département de Gynécologie Obstétrique, Faculté de Médecine, Université de Lubumbashi, Lubumbashi, République Démocratique du Congo,; 2 Institut Supérieur des Techniques Médicales de Lubumbashi, Lubumbashi, République Démocratique du Congo,; 3 Département des Sciences de Base, Faculté de Médecine, Université de Lubumbashi, Lubumbashi, République Démocratique du Congo,; 4 Faculté des Sciences Pharmaceutiques, Université de Lubumbashi, Lubumbashi, République Démocratique du Congo,; 5 Département de Gynécologie-Obstétrique, Faculté de Médecine, Université Catholique du Graben, Butembo, République Démocratique du Congo,; 6 Département d´Anesthésie et Réanimation, Faculté de Médecine, Université de Lubumbashi, Lubumbashi, République Démocratique du Congo

**Keywords:** Spermogramme, anomalies spermatiques, désir de procréation, Lubumbashi, Spermogram, sperm abnormalities, desire for procreation, Lubumbashi

## Abstract

**Introduction:**

A Lubumbashi tout comme dans les milieux huppés où les explorations de la fertilité sont bien futées, le spermogramme demeure l´analyse indispensable dans le diagnostic de l´infertilité masculine. Celle-ci est à l´origine de 40% d´infertilité du couple. Le spermogramme constitue la première étape dans l´identification des anomalies séminales. L´objectif de cette étude était de déterminer le profil épidémio-clinique et séminal de l´homme consultant pour désir de procréation à Lubumbashi.

**Méthodes:**

il s´était agi d´une étude descriptive transversale avec un volet analytique. Nous avions reçu 202 sujets de différents cabinets de consultations de Lubumbashi et dont le spermogramme avait été réalisé au laboratoire des Cliniques Universitaires de Lubumbashi du 1^er^ août 2020 au 31 juillet 2021. Les paramètres spermatiques ont été étudiés et interprétés selon les normes de l´Organisation mondiale de la Santé (OMS) (2010) avec études des facteurs associés à leur perturbation. Les analyses bivariées et multivariées avaient été réalisées. Le seuil de signification statistique avait été fixé à p < 0,05.

**Résultats:**

le profil épidémio-clinique des enquêtés était le suivant: la tranche d´âge la plus représentée était de 30 à 39 ans; l´infertilité était primaire dans 80,69% des cas; la durée du désir de paternité était de 2 ans au plus dans 44,55% des cas. Les anomalies spermatiques retrouvées étaient: l´oligozoospermie (40,09%), l´azoospermie (11,38%), l´asthénozoospermie (18,31%) et la tératozoospermie (10,39%). L´oligozoospermie était significativement associée à la varicocèle (ORa = 10,9 [3,0-39,5]; p < 0,0001), à l´infection génitale (ORa =2,7 [1,0-7,2]; p = 0,041) et à l´obésité (ORa = 2,6 [1,0-7,9]; p = 0,020) alors que l´azoospermie l´était à la cure de la hernie inguinale (ORa = 4,2 [1,0-17,2]; p = 0,049) et à la dénutrition (ORa =6,0 [1,2-29,7]; p = 0,027). L´asthénozoospermie était significativement associée à la tranche d´âge de 40 à 49 ans (ORa = 6,6 [1,2-37,4]; p = 0,034), au tabac (ORa =7,5 [2,77-21,0]; p = 0,000), à la dénutrition (ORa = 7,7 [1,0-61,9]; p = 0,045) et au surpoids (ORa =3,8 [1,3-11,5]; p = 0,019). La tératozoospermie était significativement associée au tabac (ORa = 5,6 [1,8-17,7]; p = 0,003) et au surpoids (ORa =5,3 [1,2-23,3]; p = 0,027).

**Conclusion:**

plus de la moitié des enquêtés avaient sur les trois principaux paramètres de fertilité, au moins un qui était perturbé. La numération des spermatozoïdes était le paramètre le plus atteint. L´alcool, le tabac, l´infection génitale et la malnutrition étaient les facteurs de risque les plus fréquents des anomalies observées.

## Abstract (en)

**Introduction:**

in Lubumbashi, as in upscale areas where explorations of fertility are very clever, the spermogram remains the essential analysis in the diagnosis of male infertility. This is the cause of 40% of couple infertility. The spermogram is the first step in identifying seminal abnormalities. The objective of this study was to determine the epidemiological-clinical and seminal profile of the man consulting for the desire to procreate in Lubumbashi.

**Methods:**

this was a cross-sectional study. We received 202 subjects in Lubumbashi, whose spermogram was performed from August 1^st^, 2020 to July 31^st^, 2021. The semen parameters were studied and interpreted according to WHO standards (2010) with studies of factors associated with their disturbance. Bivariate and multivariate analyzes had been carried out. The statistical significance threshold was set at p < 0.05.

**Results:**

the epidemiological-clinical profile of the respondents was as follows: the most represented age group was 30 to 39 years; infertility was primary in 80.69% of cases; the duration of the desire for paternity was 2 years at most in 44.55% of cases. The sperm abnormalities found were: oligozoospermia (40.09%), azoospermia (11.38%), asthenozoospermia (18.31%) and teratozoospermia (10.39%). Oligozoospermia was significantly associated with varicocele (ORa = 10.9 [3.0-39.5]; p < 0.0001), genital infection (ORa =2.7 [1.0-7, 2]; p = 0.041) and obesity (ORa = 2.6 [1.0-7.9]; p = 0.020) while azoospermia was the cure for inguinal hernia (ORa = 4.2 [1.0-17.2]; p = 0.049) and malnutrition (ORa =6.0 [1.2-29.7]; p = 0.027). Asthenozoospermia was significantly associated with the age group of 40 to 49 years (ORa = 6.6 [1.2-37.4]; p = 0.034), tobacco (ORa =7.5 [2.7 -21.0]; p = 0.000), undernutrition (ORa = 7.7 [1.0-61.9]; p = 0.045) and overweight (ORa =3.8 [1.3-11, 5]; p=0.019). Teratozoospermia was significantly associated with smoking (ORa = 5.6 [1.8-17.7]; p = 0.003) and overweight (ORa =5.3 [1.2-23.3]; p = 0.027).

**Conclusion:**

more than half of the respondents had, of the three main fertility parameters, at least one that was disturbed. Sperm count was the most affected parameter. Alcohol, tobacco, genital infection and malnutrition were the most common risk factors for the abnormalities observed.

## Introduction

Dans le monde, selon l´OMS, le nombre de couples infertiles est estimé entre 60 et 80 millions [1]. L´infertilité peut être attribuée à l´homme quand il existe une altération d´un ou de plusieurs paramètres spermatiques [2]. En France, un couple sur six consultera au cours de sa vie reproductive pour des difficultés à concevoir. Dans ce lot, la responsabilité de l´homme est de 20%, celle de la femme de 33% et de 39% pour les deux partenaires [3]. Au Mali, plusieurs études ont montré que la responsabilité de l´homme dans l´infertilité varie de 30 à 50% [4]. En République Démocratique du Congo (RDC), une étude menée au Kasaï avait trouvé que l´homme en était plus responsable que la femme (34,6% contre 21,4%) [5]; une autre, réalisée à Butembo, avait trouvé une prévalence de 46% d´infertilité masculine [6]. Les changements du mode de vie et climatique, l´exploitation minière et l´insalubrité avec un tas de déchets ménagers et surtout plastiques érigés sur beaucoup d´endroits dans la ville de Lubumbashi sont pourvoyeurs de la chaleur, des éléments traces métalliques (Cadmium, Arsenic, etc.) et des perturbateurs endocriniens environnementaux (phtalates, pesticides, etc.) qui ont tous des conséquences négatives sur la fertilité masculine [7-10]. Les consultations pour désir de procréation sont à en juger par notre observation, fréquentes à Lubumbashi et l´homme y étant rare, constitue un handicap pour la mise au point étiologique. Nous n´avions trouvé aucune étude à Lubumbashi dont l´intérêt portait sur le versant masculin de l´infertilité du couple. Ce faisant, l´objectif de la présente étude était d´établir un état des lieux en ce qui concerne le profil épidémio-clinique et séminal des hommes consultant pour désir de procréation dans la ville de Lubumbashi.

## Méthodes

**Cadre et type d´étude**: cette étude avait été réalisée au laboratoire des Cliniques Universitaires de Lubumbashi (CUL) et à celui du Centre Médical Stella Matutina. Il s´était agi d´une étude descriptive transversale avec un volet analytique sur une période de 12 mois soit d´août 2020 à juillet 2021.

**Participants à l´étude**: elle était constituée de tous les sujets masculins qui s´étaient présentés aux laboratoires susmentionnés pour l´analyse séminale. L´échantillonnage était exhaustif, englobant tous les cas de spermogramme réalisé à ces laboratoires durant la période d´étude. Ainsi, l´échantillon d´étude était de 202.

**Sélection et collecte des échantillons du sperme**: après une période d´abstinence de 2 à 5 jours, le recueil du sperme était effectué au laboratoire ou en dehors de l´établissement hospitalier. Dans ce dernier cas, l´échantillon nous a été apporté dans l´heure qui a suivi le recueil. L´étude du sperme n´a été entreprise qu´avec toutes les garanties que le prélèvement a été fait sans perte et dans de bonnes conditions requises à cet effet.

**Variables d´étude**: les variables indépendantes comprenaient: a) les variables sociodémographiques: âge, résidence, niveau de scolarité et la profession; les personnes suivantes étant considérées comme exerçant des professions à risque: agriculteur, jardinier, chauffeur, soudeur, peintre carrossier, électronicien, électricien, travailleur sur écran, chauffagiste, boulanger, mineur et creuseur, affuteur et technicien en radiologie; b) les antécédents personnels: consommation d´alcool, de tabac, cure de hernie inguinale, infections génitales (blennorragie, syphilis, épididymite, prostatite, orchite), oreillons, varicocèle et diabète sucré; c) le type d´infertilité; d) le lieu de prélèvement; e) le mode de prélèvement; f) le nombre de partenaires; g) la durée du désir de paternité; h) l´indice de masse corporelle (IMC). Les variables dépendantes étaient représentées par les anomalies du spermogramme. Les valeurs de référence des paramètres d´évaluation de la fertilité, correspondant au 5^e^ percentile étaient celles de l´OMS [2].

**Analyse de données**: l´encodage et la saisie des données étaient effectués grâce au logiciel Microsoft Excel 2007. L´analyse des données, qui s´est fait en utilisant le logiciel STATA 15, a compris les statistiques descriptives. Le test de Chi carré était utilisé pour comparer les proportions observées, la correction de Yates et le test exact de Fisher étant utilisés selon le contexte. Le test t de Student ou l´ANOVA (test F de Fisher-Snedecor) avait servi à comparer les moyennes, leurs équivalents non paramétriques (Tests Kruskal Wallis et Wilcoxon) étant utilisé au cas où la distribution des données n´aurait pas été normale aux tests de Kolmogorov-Smirnov et Shapiro-Wilk). La régression multivariée ainsi que l´OR ajusté à intervalle de confiance à 95% ont servi à déterminer et à évaluer les facteurs de risque éventuels. Le seuil de signification était fixé à p <0,05.

**Considérations éthiques**: la présente étude a été approuvée par le comité d´éthique médicale de l´Université de Lubumbashi (N° d´approbation: UNILU/CEM/184/2020). Le consentement libre et éclairé a été obtenu oralement de chaque participant avant l´entrevue après avoir fourni une brève explication concernant l´étude. Chaque participant a également été informé qu´il a le droit de refuser ou de retirer sa participation à tout moment et qu´aucun préjudice ne sera imposé à la suite de sa participation ou de son refus. Lors de la collecte des données, les identifiants personnels tels que le nom et les numéros de téléphone des participants à l´étude n´avaient jamais été enregistrés. Les données recueillies ont été gardées confidentielles et utilisées uniquement aux fins de l´étude.

## Résultats

**Profil épidémio-clinique des patients**: l´âge moyen des enquêtés était de 36,2 ± 6,3 ans (extrêmes: 25 et 65 ans); plus de la moitié (58,4%) d´entre eux avaient un âge compris entre 30 et 39 ans. Quant à la profession qu´ils exerçaient, il s´est avéré que 20,3% des cas pratiquaient une profession à risque. Plus de 4 enquêtés sur 10 (47,03%) consommaient de l´alcool et 22,78% du tabac; 25,25% avaient un antécédent d´infection génitale; 8,91% avaient bénéficié d´une cure de hernie inguinale; 5,94% avaient fait des oreillons et 9,41% avaient présenté une varicocèle. Cent soixante-trois (80,69%) enquêtés présentaient une infertilité primaire; 4,46% d´entre eux avaient 2 partenaires ou plus. Un désir de paternité d´au plus de deux ans était signalé par 44,55% d´entre eux. Dans 55,45% des cas, l´indice de masse corporelle était anormal: 34,16% des enquêtés étaient en surpoids; 16,34% étaient obèses tandis que 4,95 % étaient dénutris.

**Profil séminal des patients**: le pH du sperme était acide chez 5,9 % des enquêtés et 9,4% de ceux-ci avaient une hypospermie. Les anomalies microscopiques observées étaient les suivantes: l´oligozoospermie (21,8%), l´azoospermie (11,4%), l´asthénozoospermie (2,5%), la tératozoospermie (1%), l´oligotératozoospermie (2,5%), l´oligoasthénozoospermie (8,9%) et l´oligoasthénotératozoospermie (6,9%).

**Facteurs associés aux anomalies séminales**: les sujets âgés de 50 ans et plus, avaient présenté la proportion la plus élevée des cas d´oligozoospermie (42,9%) par rapport aux autres tranches d´âge sans que la différence soit statistiquement significative (Tableau 1, Tableau 2). En ce qui concerne l´asthénozoospermie, la tranche d´âge de 40-49 ans avait présenté la proportion la plus élevée (24, 40%) et l´analyse multivariée avait mis en évidence une différence significative, la tranche d´âge de 40-49 ans ayant présenté un risque près de sept fois supérieur par rapport à la tranche d´âge de 20-29 ans, dont la proportion des cas était de 13% (Tableau 3). Après régression logistique, aucune anomalie des paramètres spermatiques n´a été liée significativement aux professions à risque (Tableau 1, Tableau 2, Tableau 3, Tableau 4). Dans notre série, 47,0% des enquêtés consommaient l´alcool mais à l´analyse multivariée, l´alcool n´était lié à aucune anomalie des paramètres spermatiques (Tableau 1, Tableau 2, Tableau 3, Tableau 4). Les sujets qui fumaient ont été retrouvés dans 22,8% des cas et à l´analyse multivariée, le tabac s´était révélé un déterminant de l´altération de la mobilité (ORa= 7,5[2,7-21,0]; p = 0,034) et de la morphologie des spermatozoïdes (ORa= 5,6 [1,8-17,7]; p= 0,003) avec un risque respectif d´environ huit et six fois plus élevé (Tableau 1, Tableau 2, Tableau 3, Tableau 4). La cure de la hernie inguinale a été retenue parmi les déterminants de l´azoospermie (ORa = 4,2[1,0-17,2]; p= 0,049), le risque étant alors multiplié par quatre (Tableau 1). La varicocèle a été enregistrée chez 9,4% des enquêtés et s´est révélée être un déterminant de l´oligozoospermie avec un risque multiplié par onze (Tableau 2). Dans notre série, le diabète sucré a été trouvé chez 7,4% des enquêtés et n´était significativement associé à aucune anomalie spermatique après analyse multivariée (Tableau 1). Il ressort de notre étude que 34,2% des enquêtés étaient en surpoids, 16,3% obèses et 5,0% dénutris. L´azoospermie était significativement associée à la dénutrition, même à l´issue de l´analyse multivariée (ORa =6,0 [1,2-29,7]; p=0,027), l´état de dénutrition s´étant ainsi avéré l´un des déterminants de l´azoospermie dans notre série avec un risque multiplié par six (Tableau 1).

**Tableau 1:** âge, profession, antécédents personnels, IMC et azoospermie

Variable		Total	Azoospermie				Analyse bivariée		Analyse multivariée		
		(N=202)	Oui (n=23)		Non (n=179)		OR brut [ICà95%]	p	OR ajusté	[IC à 95%]	p
**Âge (ans)**	20-29	26(12,9%)	3	11,50%	23	88,50%	1		1,0		
	30-39	118(58,4%)	10	8,50%	108	91,50%	0,7 [0,2-2,8]	0,908	1,1	[0,2 5,5]	0,889
	40-49	51(25,3%)	10	19,60%	41	80,40%	1,9 [0,4-11,5]	0,567	3,3	[0,6 18,6]	0,178
	≥50	7(3,5%)	0	0,00%	7	100,00%	0,0 [0,0-9,5]	1	1,0		
**Profession**	À risque	47(20,3%)	3	6,40%	44	93,60%	0,5 [0,1-1,7]	0,298	10,3	[0,1 1,4]	0,131
	Sans risque	155	20	12,90%	135	87,10%	1		1		
**Alcool**	Non	107	11	10,30%	96	89,70%	1		1		
	Oui	95(47,0%)	12	12,60%	83	87,40%	0,8 [0,3-1,9]	0,762	0,7	[0,2 2,0]	0,514
**Tabagisme**	Non	156	13	8,30%	143	91,70%	1		1		
	Oui	45(22,8%)	10	21,70%	36	78,30%	**3,1 [1,2-7,5]**	**0,024**	3,3	[0,9 11,5]	0,067
**Hernie inguinale**	Non	184	18	9,80%	166	90,20%	1		1		
	Oui	18(25,3%)	5	27,80%	13	72,20%	**3,5 [1,1-11,1]**	**0,022**	**4,2**	**[1,0 17,2]**	**0,049**
**Varicocèle**	Non	183	20	10,90%	163	89,10%	1		1		
	Oui	19(9,4%)	3	15,80%	16	84,20%	1,5 [0,3-6,0]	0,46	0,6	[0,1 3,4]	0,565
**Infection génitale**	Non	151	13	8,60%	138	91,40%	1		1		
	Oui	51(25,3%)	10	19,60%	41	80,40%	**2,6 [1,1-6,3]**	**0,032**	2,1	[0,6 6,9]	0,233
**Oreillons**	Non	190	21	11,10%	169	89,00%	1		1		
	Oui	12(5,9%)	2	16,70%	10	83,30%	1,6 [0,2-8,3]	0,631	4	[0,8 20,9]	0,103
**Diabète**	Non	187	18	9,60%	169	90,40%	1		1		
**sucré**	Oui	15(7,4%)	5	33,30%	10	66,70%	**4,7 [1,4-15,3]**	**0,018**	0,6	[0,1 4,1]	0,572
**IMC** (kg/m^2^)	<18,5	10(5,0%)	4	40,00%	6	60,00%	**6,0 [1,4-25,3]**	**0,007**	**6**	**[1,2 29,7]**	**0,027**
	18,5-24,9	90(44,6%)	9	10,00%	81	90,00%	1		1		
	25,0-29,9	69(34,2%)	6	8,70%	63	91,30%	0,9 [0,3-2,5]	0,996	0,7	[0,2 2,5]	0,606
	≥30,0	33(16,3%)	4	12,10%	29	87,90%	1,2 [0,4-4,3]	0,993	1	[0,2 4,3]	0,948

**Tableau 2:** âge, profession, antécédents personnels, IMC et oligozoospermie

Variable		Total	Oligozoospermie				Analyse bivariée		Analyse multivariée		
		(N=179)	Oui (n=44)		Non (n=135)		OR brut[ICà95%]	p	OR ajusté	[IC à 95%]	p
Âge(ans)	20-29	23	6	26,1%	17	73,9%	1,0		1,0		
	30-39	108	28	25,9%	80	74,1%	1,0 [0,4-2,8]	1,000	1,0	[0,3-3,3]	0,935
	40-49	41	7	17,1%	34	82,9%	0,6 [0,2-2,0]	0,592	0,3	[0,1-1,3]	0,097
	≥50	7	3	42,9%	4	57,1%	2,1 [0,2-16,8]	0,640	1,8	[0,3-12,5]	0,556
Profession	Sans risque	135	27	20,0%	108	80,0%	1,0		1,0		
	À risque	44	17	38,6%	27	61,4%	2,5 [1,2-5,3]	0,022	1,9	[0,8-4,7]	0,154
Alcool	Non	96	21	21,9%	75	78,1%	1,0		1,0		
	Oui	83	23	27,7%	60	72,3%	0,7 [0,4-1,4]	0,465	0,9	[0,4-2,2]	0,877
Tabagisme	Non	143	35	24,5%	108	75,5%	1,0		1,0		
	Oui	36	9	25,0%	27	75,0%	1,0 [0,4-2,4]	1,000	0,4	[0,1-1,1]	0,061
Hernie inguinale	Non	166	41	24,7%	125	75,3%	1,0		1,0		
	Oui	13	3	23,1%	10	76,9%	0,9 [0,2-3,8]	1,000	1,1	[0,2-4,8]	0,921
Varicocèle	Non	163	34	20,9%	129	79,1%	1,0		1,0		
	Oui	16	10	62,5%	6	37,5%	6,3[2,1-18,6]	0,001	10,9	[3,0-39,5]	0,000
Infection génitale	Non	138	27	19,6%	111	80,4%	1,0		1,0		
	Oui	41	17	41,5%	24	58,5%	2,9[1,4-6,2]	0,008	2,7	[1,0-7,2]	0,041
Diabète sucré	Non	169	39	23,1%	130	76,9%	1,0		1,0		
	Oui	10	5	50,0%	5	50,0%	3,3 [0,9-12,1]	0,067	2,4	[0,5-11,1]	0,277
Oreillons	Non	169	40	23,7%	129	76,3%	1,0		1,0		
	Oui	10	4	40,0%	6	60,0%	2,1 [0,4-9,5]	0,264	1,1	[0,2-6,1]	0,888
IMC(kg/m^2^ )	<18,5	6	1	16,7%	5	83,3%	1,0 [0,0-9,6]	1,000	1,1	[0,1-11,7]	0,929
	18,5-24,9	81	14	17,3%	67	82,7%	1,0		1,0		
	25,0-29,9	63	20	31,8%	43	68,3%	2,2 [0,9-5,3]	0,067	2,7	[1,0-6,8]	0,041
	≥30,0	29	9	31,0%	20	69,0%	2,1[0,8-5,7]	0,195	2,6	[1,0-7,9]	0,020

**Tableau 3:** âge, profession, antécédents personnels, IMC et asthénozoospermie

Variable		Total	Asthénozoospermie				Analyse bivariée		Analyse multivariée		
		(N=179)	Oui(n=37)		Non(n=142)		OR brut [IC à95%]	p	OR ajusté	[IC à 95%]	p
**Âge (ans)**	20-29	23	3	13,00%	20	87,00%	1		1		
	30-39	108	23	21,30%	85	78,70%	1,8 [0,5-10,3]	0,565	3,8	[0,8-18,6]	0,095
	40-49	41	10	24,40%	31	75,60%	2,1[0,5-13,5]	0,346	**6,6**	**[1,2-37,4]**	**0,034**
	≥50	7	1	14,30%	6	85,70%	1,1 [0,0-17,1]	1	0,9	[0,0-25,7]	0,955
**Profession**	À risque	44	13	29,60%	31	70,50%	1,9 [0,9-4,2]	0,144	1,3	[0,5-3,6]	0,609
	Sans risque	135	24	17,80%	111	82,20%	1		1		
**Alcool**	Non	96	13	13,50%	83	86,50%	1		1		
	Oui	83	24	28,90%	59	71,10%	**2,6 [1,2-5,5]**	**0,018**	1,5	[0,6-3,8]	0,433
**Tabagisme**	Non	143	18	12,60%	125	87,40%	1		1		
	Oui	36	19	52,80%	17	47,20%	**7,8 [3,4-17,6]**	**0,000**	**7,5**	**[2,7-21,0]**	**0,000**
**Hernie inguinale**	Non	166	33	19,90%	133	80,10%	1		1		
	Oui	13	4	30,80%	9	69,20%	1,8 [0,5-6,2]	0,474	3,9	[0,8-20,1]	0,099
**Varicocèle**	Non	163	35	21,50%	128	78,50%	1		1		
	Oui	16	2	12,50%	14	87,50%	0,5 [0,1-2,5]	0,53	0,2	[0,0-1,3]	0,083
**Infection génitale**	Non	138	22	15,90%	116	84,10%	1		1		
	Oui	41	15	36,60%	26	63,40%	**3,0 [1,4-6,6]**	**0,008**	1,6	[0,6-4,4]	0,339
**Oreillons**	Non	169	33	19,50%	136	80,50%	1		1		
	Oui	10	4	40,00%	6	60,00%	2,7 [0,5-12,3]	0,219	1,6	[0,3-9,4]	0,602
**Diabète**	Non	169	33	19,50%	136	80,50%	1		1		
**sucré**	Oui	10	4	40,00%	6	60,00%	2,7[0,5-12,3]	0,219	1	[0,2-5,6]	0,972
**IMC**(kg/m^2^)	<18,5	6	3	50,00%	3	50,00%	**10,0 [1,1-90,4]**	**0,002**	**7,7**	**[1,0-61,9]**	**0,045**
	18,5-24,9	81	7	8,60%	74	91,40%	1		1		
	25,0-29,9	63	19	30,20%	44	69,80%	**4,6 [1,8-11,7]**	**0,002**	**3,8**	**[1,3-11,5]**	**0,019**
	≥30,0	29	8	27,60%	21	72,40%	**4,0 [1,3-12,4]**	**0,025**	3,1	[0,8-11,3]	0,083

**Tableau 4:** âge, profession, antécédents personnels, IMC et tératozoospermie

Variable		Total	Tératozoospermie				Analyse bivariée		Analyse multivariée		
		(N=179)	Oui (n=21)		Non(n=159)		OR brut [IC à 95%]	p	OR ajusté	[IC 95%]	p
**Âge (ans)**	20-29	23	3	13,00%	20	87,00%	1		1		
	30-39	108	10	9,30%	98	90,70%	0,7[0,2-4,2]	0,698	1	[0,2-4,8]	0,969
	40-49	41	7	17,10%	34	82,90%	1,4 [0,3-9,1]	1	2,7	[0,5-16,3]	0,269
	≥50	7	1	14,30%	6	85,70%	1,1 [0,0-17,1]	1	0,9	[0,0-20,4]	0,969
**Profession**	À risque	44	7	15,90%	37	84,10%	1,6[0,6-4,4]	0,47	1,2	[0,4-3,9 ]	0,746
	Sans risque	135	14	10,40%	121	89,60%	1		1		
**Alcool**	Non	96	6	6,30%	90	93,80%	1		1		
	Oui	83	15	18,10%	68	81,90%	**3,3 [1,2-9,0]**	**0,025**	2,5	[0,8-8,4]	0,129
**Tabagisme**	Non	143	9	6,30%	134	93,80%	1		1		
	Oui	36	12	33,30%	24	66,70%	**7,4 [2,8-19,6]**	**0**	**5,6**	**[1,8-17,7]**	**0,003**
**Hernie inguinale**	Non	166	19	11,50%	147	88,60%	1		1		
	Oui	13	2	15,40%	11	84,60%	1,4 [0,3-6,8]	0,672	2,4	[0,3-17,3]	0,374
**Varicocèle**	Non	163	19	11,70%	144	88,30%	1		1		
	Oui	16	2	12,50%	14	87,50%	1,1 [0,2-5,1]	1	0,6	[0,1-4,0]	0,605
**Infection génitale**	Non	138	13	9,40%	125	90,60%	1		1		
	Oui	41	8	19,50%	33	80,50%	2,3 [0,9-6,1]	0,137	0,9	[0,3-2,8]	0,8
**Oreillons**	Non	169	18	10,70%	151	89,40%	1		1		
	Oui	10	3	30,00%	7	70,00%	3,6 [0,9-15,1]	0,179	1,5	[0,2-10,4]	0,685
**Diabète**	Non	169	18	10,70%	151	89,40%	1		1		
**sucré**	Oui	10	3	30,00%	7	70,00%	3,6 [0,9-15,1]	0,179	1,4	[0,2-8,3]	0,726
**IMC** (kg/m^2^)	<18,5	6	1	16,70%	5	83,30%	5,1 [0,1-79,0]	0,25	3,3	[0,2-52,9]	0,407
	18,5-24,9	81	3	3,70%	78	96,30%	1		1		
	25,0-29,9	63	13	20,60%	50	79,40%	**6,8 [1,7-38,8]**	**0,002**	**5,3**	**[1,2-23,3]**	**0,027**
	≥30,0	29	4	13,80%	25	86,20%	4,1 [0,7-30,2]	0,075	2,6	[0,5-14,5]	0,281

## Discussion

Dans notre série, le pH du sperme était acide chez 5,94% des enquêtés. Mukendi *et al*. ont trouvé un pH acide chez 32% de leur échantillon d´étude, exposés et non exposés confondus [7]. Cette grande différence pourrait s´expliquer, d´une part, par le fait que dans notre travail l´acidité était considérée quand le pH était inférieur à 7, conséquence de l´utilisation d´une bandelette avec précision de 0,5, et d´autre part, par le fait que la fréquence élevée observée dans cette dernière étude était due à la présence des exposés avec une forte concentration en Cadmium et Arsenic, ces derniers étant associés à l´acidification du sperme et présentant un fort pouvoir reprotoxique. Notre étude a trouvé l´oligozoospermie non combinée à d´autres anomalies comme le diagnostic le plus fréquent (21,78%), suivie de l´azoospermie (11,38%) et de l´oligoasthénozoospermie (8,91%). Contrairement à nous, Kalenga *et al*. avaient trouvé l´oligoasthénozoospermie (56,2%) comme diagnostic le plus fréquent de leur étude réalisée dans la région centrale de la RDC [5] et Matumo *et al*., à l´Est de la RDC, avaient trouvé en tête l´oligoasthénotératozoospermie (OATS) dans 44,8% des cas, suivie de l´oligoasthénozoospermie dans 36,5% et enfin l´asthénotératozoospermie dans 5,2% des cas [6]. Il sied de noter que les prévalences d´anomalies dans cette dernière étude étaient élevées parce que son échantillon n´avait tenu compte que des sujets dont le spermogramme portait une anomalie. Dans une étude de prévalence au Maroc, Frick *et al*. ont trouvé une prévalence de 16,4% des cas d´azoospermie [1]. Dans la littérature, il est indiqué que la prévalence de l´azoospermie est estimée à 10 à 20% des hommes infertiles [11]. C´est le cas dans notre série où elle était de 11,38%.

Dans notre étude, près de six enquêtés sur 10 (58,42 %) étaient dans la tranche d´âge de 30 à 39 ans (Tableau 1). C´est à cet âge effectivement que les hommes commencent à prendre au sérieux le besoin d´enfants et s´impliquent aussi dans la recherche des solutions à leur problème d´infertilité. Jusque-là, ils pensaient que c´est la femme qui serait seule responsable et qu´elle serait seule concernée par la recherche des solutions et subséquemment par le traitement de l´infertilité du couple. D´autres auteurs ont trouvé des résultats similaires aux nôtres [12,13]. Le désir d´enfant est exprimé même à un âge très avancé. Cet âge avancé reflète d´une part l´âge avancé du mariage mais également le barrage psychologique des hommes par rapport à leur infertilité, ce qui justifierait les consultations tardives. De plus, les contraintes sociales et professionnelles actuelles ont conduit à un changement du style de vie des populations et un retard du désir de procréation [14]. Plusieurs études prouvent l´effet du vieillissement sur tous les paramètres spermatiques et ce au-delà de 45 ans [13]. Alors que dans notre étude, seule la mobilité des spermatozoïdes (ORa = 6,6 [1,2-37,4] ; p = 0,034) s´est révélée significativement affectée. Dans notre série 47,03% des enquêtés consommaient l´alcool. La fréquence des consommateurs d´alcool de notre série est sensiblement supérieure à celles rapportées par Ousmane, soit 7% [4] et Ouattara *et al*. soit 4% [15]. Ces faibles taux de consommation d´alcool enregistrés tout au long des différentes études faites au Mali s´expliquent par le fait que la société est à 90% musulmane et l´islam interdit la consommation d´alcool. Quoique notre étude n´aie pas trouvé de lien entre les anomalies spermatiques et l´éthanol, celui-ci agirait sur l´axe hypothalamo-hypophysaire en désynchronisant la sécrétion pulsatile de la *Gonadotropin hormone-releasing hormone* (GnRH) et en générant alors un hypogonadisme hypogonadotrope partiel [16].

Le tabagisme a été retrouvé dans notre série dans 22,78% des cas. À l´analyse multivariée, le tabac s´était révélé un déterminant de l´altération de la mobilité (ORa= 7,5 [2,7-21,0]; p = 0,034) et de la morphologie des spermatozoïdes (ORa= 5,6 [,8- 17,7]; p= 0,003). Mohamed F. *et al*. avaient retrouvé le tabac dans 28,6% des cas, ce facteur étant le plus représenté dans leur série et responsable des perturbations des paramètres spermatiques sans que celles-ci soient statistiquement significatives [1]. L´effet délétère du tabac sur les paramètres spermatiques est le fait de la perturbation des taux hormonaux par le truchement de la nicotine ainsi que d´autres produits chimiques [17]. Un certain nombre de composants présents dans la cigarette entraîneraient une tératozoospermie plus importante que chez les non-fumeurs et seraient associés à une augmentation du risque de dommages causés à l´ADN de la lignée germinale via le stress oxydatif, produit par la fumée de cigarette [18]. Il modifie ainsi la mobilité et la morphologie des spermatozoïdes causant probablement une diminution du pouvoir fécondant de ceux-ci [19]. L´antécédent de hernie inguinale a été enregistrée dans 8,91% des enquêtés. Cette prévalence est supérieure à celle de la série de Mohamed F. *et al*. qui était de 5,4% [1]. Nos résultats montrent que la cure de la hernie inguinale a été un déterminant de l´azoospermie (ORa = 4,2[1,0-17,2]; p= 0,049). L´obstruction des canaux déférents due à une cure de hernie inguinale représenterait une cause iatrogène possible d´infertilité masculine [20].

La varicocèle a été enregistrée dans 9,41% des enquêtés de notre série. L´oligozoospermie en était l´anomalie spermatique qui a présenté une corrélation significative (ORa ajusté = 10,9[3,0-39,5]; p = 0,000). La faible concentration de spermatozoïdes est attribuée par certains chercheurs à l´apoptose élevée des cellules germinales habituellement observée chez ces hommes [21]. Nous avions enregistré 80,69 % de cas d´infertilité primaire. Ce taux est variable selon les séries et se situe entre 57,4 et 82% [22]. Il ressort de notre étude que 34,16% des enquêtés étaient en surpoids, 16,34% étaient obèses et 4,95% étaient dénutris. L´azoospermie était significativement associée à la dénutrition (ORa =6,0 [1,2-29,7]; p=0,027) (Tableau 1). La dénutrition serait responsable d´une azoospermie sécrétoire par carence en gonadotrophines et androgènes. Cette supposition suggère d´autres études dans l´avenir pour en saisir la portée.

## Conclusion

Il ressort des résultats de cette étude que la grande majorité des enquêtés présentait une infertilité masculine primaire, la consultation intervenant tardivement, c´est-à-dire à un âge frisant la quarantaine. Les anomalies séminales les plus fréquentes étaient: l´oligozoospermie, l´azoospermie, l´oligoasthénozoospermie et l´oligoasthénotératozoospermie. L´âge, l´indice de masse corporelle anormal, le tabagisme ainsi que les antécédents de varicocèle et de cure de hernie inguinale s´étaient révélés être les déterminants de l´une ou l´autre des anomalies séminales. Ainsi, la prise en compte de l´importance et des caractéristiques de l´infertilité masculine devrait être sérieusement considérée dans la prise en charge de l´infertilité du couple. Par conséquent, une stratégie d´approche idoine devrait être mise en œuvre pour optimiser cette prise en charge en vue de l´amélioration de la santé de la reproduction dans notre environnement.

### Etat des connaissances sur le sujet

- *Les consultations pour désir de procréation sont légion en République Démocratique du Congo;*
- *L´homme y est souvent absent, pourtant il en est responsable au même titre que la femme;*
- *L´âge, la profession, le tabac, l´alcool et l´obésité perturbent les paramètres spermatiques*.

### Contribution de notre étude à la connaissance

- *Cette étude a décrit le profil épidémio-clinique et séminal des hommes des couples infertiles à Lubumbashi, en République Démocratique du Congo;*
- *Elle a trouvé que presque la moitié de l´échantillon avait au moins un paramètre spermatique perturbé;*
- *Elle a prouvé que l´âge, le tabac, la cure de la hernie inguinale, la varicocèle, les infections génitales, la dénutrition, le surpoids et l´obésité étaient des facteurs perturbateurs des paramètres spermatiques*.
